# Oblique lumbar interbody fusion—address to an anatomical challenge: a case report

**DOI:** 10.3389/fsurg.2025.1621533

**Published:** 2025-08-20

**Authors:** Yuxin Meng, Shangju Gao, Fantao Meng, Wenyi Li, Yanwang Niu, Jingchao Wei

**Affiliations:** ^1^ Hebei North University, Zhangjiakou, Hebei, China; ^2^ Department of Orthopedics, Hebei General Hospital, Shijiazhuang, Hebei, China

**Keywords:** oblique lumbar interbody fusion, left-sided inferior vena cava, vascular anomaly, lateral screw-rod fixation, lumbar degenerative disease

## Abstract

Oblique lumbar interbody fusion (OLIF) is a minimally invasive technique for the treatment of degenerative diseases of the lumbar spine, and the left operative window is always used to avoid the inferior vena cava (IVC). However, in cases with anatomical variations—particularly vascular anomalies, which most significantly impact surgical approaches—the right retroperitoneal approach may serve as a preferable alternative. This case report describes a 59-year-old man with lumbar instability and a rare left-sided IVC who underwent OLIF via a right approach. Preoperative imaging of this patient showed an isolated left IVC. The procedure was performed through a right-sided surgical corridor bounded medially by the abdominal aorta(AA) and laterally by the right psoas major muscle. The approach was supplemented with lateral screw-rod instrumentation to maintain stability. The patient's neurological function improved significantly after surgery, and the surgical approach proved to be feasible while maintaining biomechanical stability while avoiding vascular risk. This case highlights the importance of vascular evaluation before OLIF. Especially for the rare left-sided IVC, OLIF via right approach can effectively improve safety.

## Introduction

The choice of surgical approach for each spinal fusion technique is governed by unique anatomical considerations. In the lumbar region, the abdominal aorta (AA) and inferior vena cava (IVC) run anterolaterally along the vertebral column, positioned left-anterior and right-anterior to the vertebral bodies, respectively. The bilateral psoas muscles originate from the lateral aspects of the lumbar vertebrae and their transverse processes. A natural anatomical corridor exists between the psoas muscle and the AA, which the oblique lumbar interbody fusion (OLIF) technique utilizes, accessing the target intervertebral disc through a left abdominal incision while navigating this avascular plane. OLIF represents a minimally invasive fusion technique that utilizes an oblique lateral approach. Compared to posterior approaches, this technique preserves the lamina, paraspinal muscles, and facet joints, consequently reducing approach-related complications (1). These advantages have established OLIF as a preferred surgical option for degenerative lumbar pathologies. Previous studies have demonstrated significant postoperative improvements in thecal sac cross-sectional area, foraminal dimensions, and neurological function following OLIF procedures (2).

However, the retroperitoneal surgical channel passes by major blood vessels, which carries a potential risk of vascular-related complications that can result in catastrophic iatrogenic injury. And the incidence of vascular-related complications was reported as about 0.37% (3). Therefore, it is necessary to select an appropriate operative window to effectively prevent the occurrence of vascular-related complications (4, 5). The IVC is more susceptible to intraoperative injury due to its thin wall and low elasticity. The IVC originates at the L4-L5 vertebral level through the confluence of the left and right common iliac veins (CIV), located anterolateral to the right side of the spinal column. From this junction, it ascends cephalad along the right anterolateral aspect of the vertebral bodies, maintaining a consistent parallel course with the AA throughout its thoracic trajectory. Due to its thin-walled structure and low elasticity, the IVC demonstrates particular vulnerability to intraoperative injury. Therefore, the conventional left-sided OLIF approach exploits the natural retroperitoneal plane between the abdominal aorta (medially) and psoas major (laterally). This strategic selection reduces the risk of injuring the thin-walled IVC. Notably, anatomical studies have identified left-sided IVC variants in 0.2%−0.5% of asymptomatic individuals (6, 7). For these patients, a right abdominal approach offers superior anatomical compatibility (8).

This case report describes a successful OLIF performed via a right retroperitoneal approach in a patient with degenerative lumbar disease and left-sided IVC anomaly. It should be emphasized that the left and right retroperitoneal approaches are not anatomical mirror images. Significant variations exist in the lumbar venous plexus distribution, sympathetic nerve relationships, and relative spatial positioning between vascular structures and the spine. Despite the anatomical differences compared to the standard left-sided approach, the procedure achieved favorable clinical outcomes while effectively preventing vascular injury. Key to this case were: (1) detailed preoperative vascular evaluation and (2) strategic utilization of the right surgical window, which collectively enabled safe OLIF execution despite the vascular anomaly.

## Case presentation

**Figure 1:**
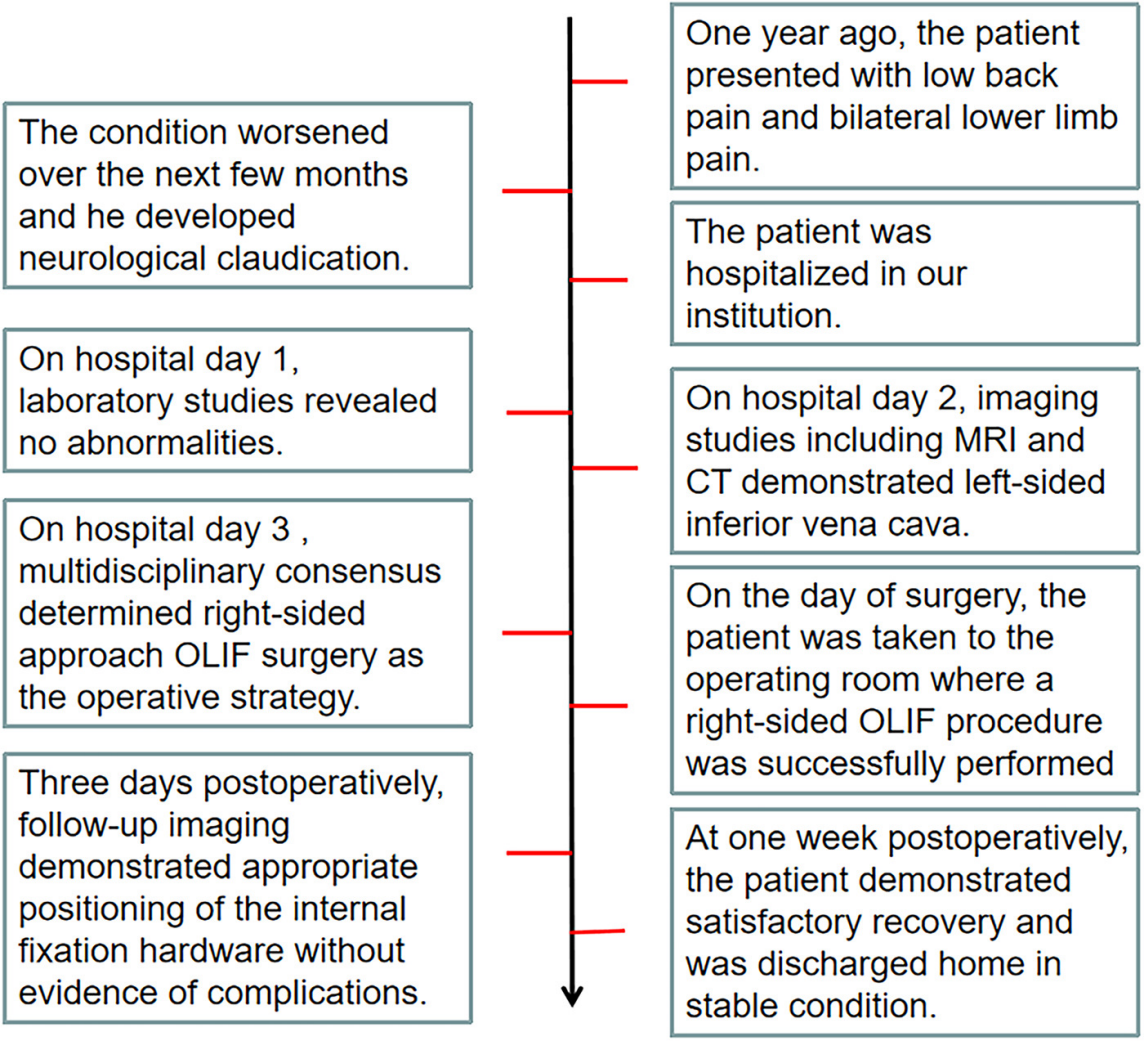
The diagram illustrates the entire process of the patient from the onset of the disease, through diagnosis and treatment, to full recovery.

A 59-year-old male patient presented with progressively worsening low back pain and bilateral lower limb pain and numbness despite one year of unsuccessful conservative therapy and pain management. The condition worsened over the next few months, and he developed neurological claudication (Figure 1). The visual analog scale (VAS) score for low back pain was 7 points, and the VAS score for lower extremity pain was 5 points, with an Oswestry Disability Index (ODI) of 58.

On physical examination, lumbar spine flexion and extension were limited, pain was caused by tapping and light pressure, the foraminal compression test was positive, and the bilateral straight leg elevation test was positive by 50°. The complete blood count (CBC) test showed a WBC count of 9.97 × 10⁹/L, an RBC count of 5.4 × 10¹²/L, hemoglobin of 14.8 g/dl, and a platelet count of 406 × 10⁹/L. Hepatorenal function tests indicated a total protein of 68.9 g/L, albumin of 32.8 g/L, total bilirubin of 7.3 μmol/L, AST of 11.1 U/L, ALT of 12.01 U/L, urea of 6.3 mmol/L, serum creatinine (Cr) of 67.0 μmol/L, and a GFR of 99.98 ml/min. Bone mineral density and metabolism were evaluated, revealing a 25-hydroxyvitamin D level of 11.24 ng/ml. Bone mineral density was assessed using dual-energy x-ray absorptiometry (DXA). The lowest value of the spine was 0.846 g/cm². The lowest value of the ulnar radius was 0.650 g/cm². The lowest value of the hip joint was 0.546 g/cm². It belongs to osteoporosis. Urine analysis and coagulation five showed no significant abnormalities.

**Figure 2:**
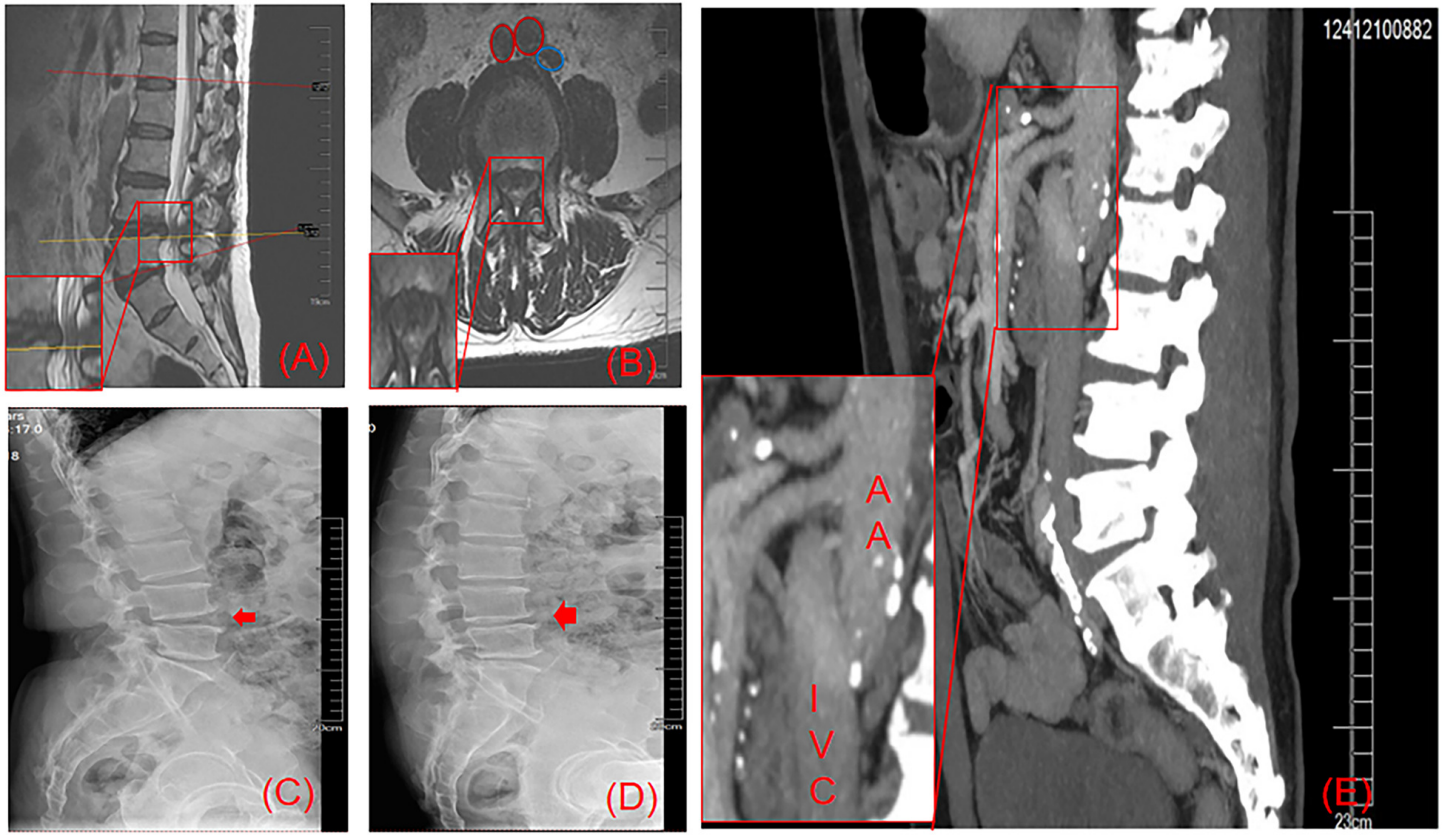
As shown in the magnified sagittal **(A)** and axial **(B)** T2-weighted MRI views, a massive L4-5 disc herniation with significant spinal canal stenosis and neural compression is observed. Figure B demonstrates the left-sided IVC (blue circle) and the bifurcated AA (red circle) at the L4-5 level. Comparative dynamic radiographs **(C)**: hyperextension; **(D)** hyperflexion) reveal 11° of angular change between the L4-5 endplates. **(E)** (IVC venography) confirms the IVC's left-anterior course along the spine, crossing ventral to the aorta at the L1 inferior endplate.

Magnetic resonance imaging (MRI) (Figures 2A,B) showed a herniated disc at the L4-L5 level, resulting in an abnormally narrow spinal canal. Unlike the common case, the MRI of the patient's lumbar spine revealed that the IVC was located on the left side of the AA. The L4-L5 intervertebral disc space was unstable with an angle variation of 11° on the hyperextension lateral radiographs and the flexion lateral radiographs (Figures 2C,D). We confirmed that the patient had a left-sided IVC by CT venography. The imaging (Figure 2E) demonstrated that the IVC, formed by the union of the left and right CIV, arises superior and to the left of the aorta. At the level of approximately the L1-2 intervertebral space, where the left and right renal veins converge, it sequentially crosses the midline of the spine and the AA, eventually coursing along the left side of the AA. This is consistent with the left-sided IVC described in the literature (9).

### Diagnosis

1.Grade I L4-L5 spondylolisthesis (anterior displacement of L4 vertebral body relative to L5, Meyerding classification); 2. L4-L5 lumbar spinal stenosis secondary to:Central disc protrusion

### Surgical plan

For this lumbar fusion case, we recommend the oblique lumbar interbody fusion (OLIF) procedure with lateral fixation to optimally balance the patient's clinical needs with minimally invasive objectives.

### Surgical procedure

**Figure 3:**
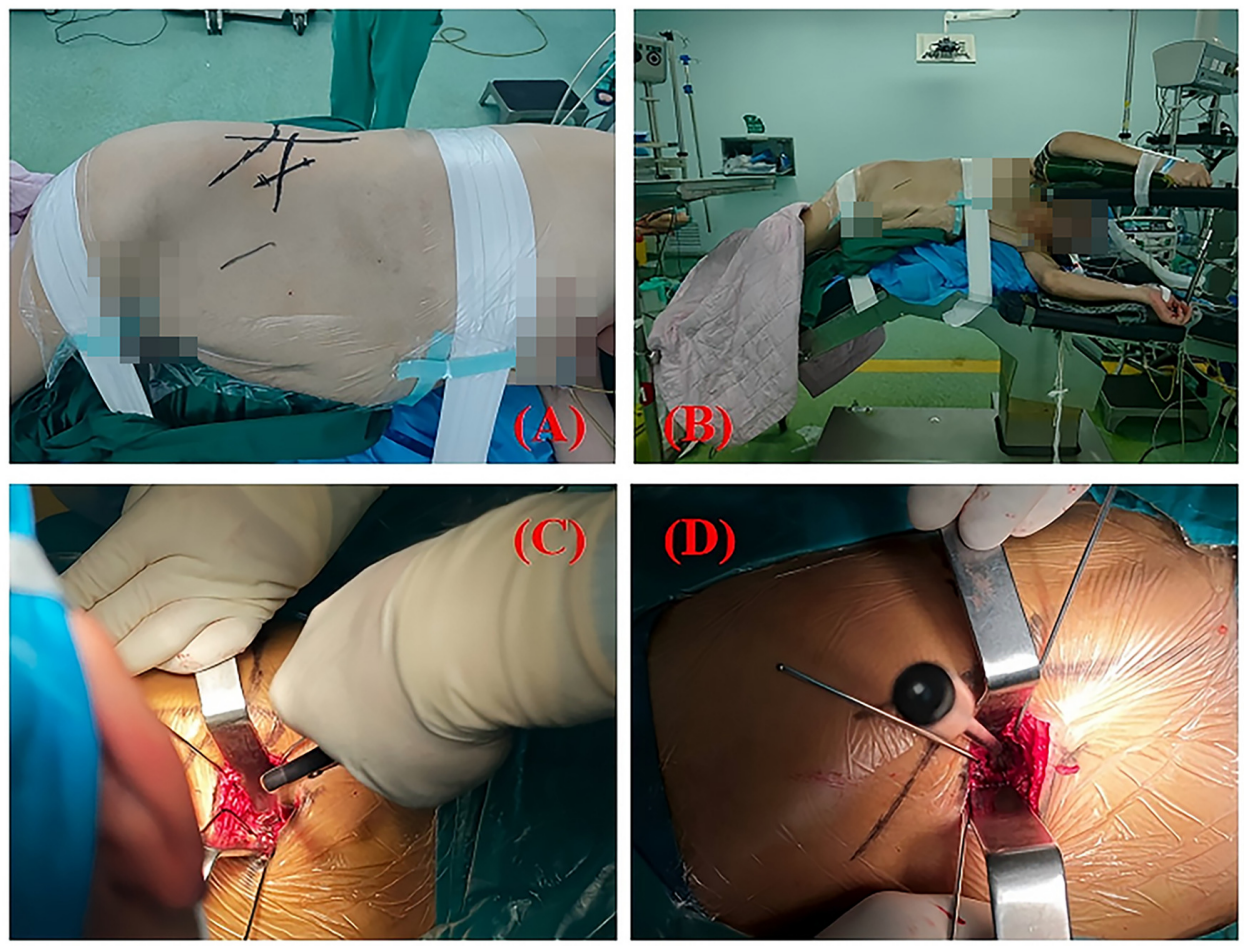
**(A,B)** demonstrates the standardized OLIF positioning, with the patient in left lateral decubitus and hips elevated. Skin markings are visible for surgical planning. **(C)** demonstrates the surgeon performing discectomy using a pituitary rongeur, while **(D)** shows the trial cage being inserted into the intervertebral space.

After successful anesthesia, the patient was placed in the left lateral decubitus position. Standard chest and iliac supports were applied, with additional bolsters placed between the legs (padded with blankets) and a protective donut-shaped pad under the dependent fibular head (Figure 3). The operating table was flexed at the lumbar bridge to level the surgical-side flank with the thorax and pelvis, thereby increasing the distance between the left costal margin and iliac crest to optimize surgical access. Under fluoroscopic guidance, the anterior superior iliac spine (ASIS) and iliac crest were identified and marked, followed by localization and marking of the target L4-5 intervertebral disc space.

**Figure 4:**
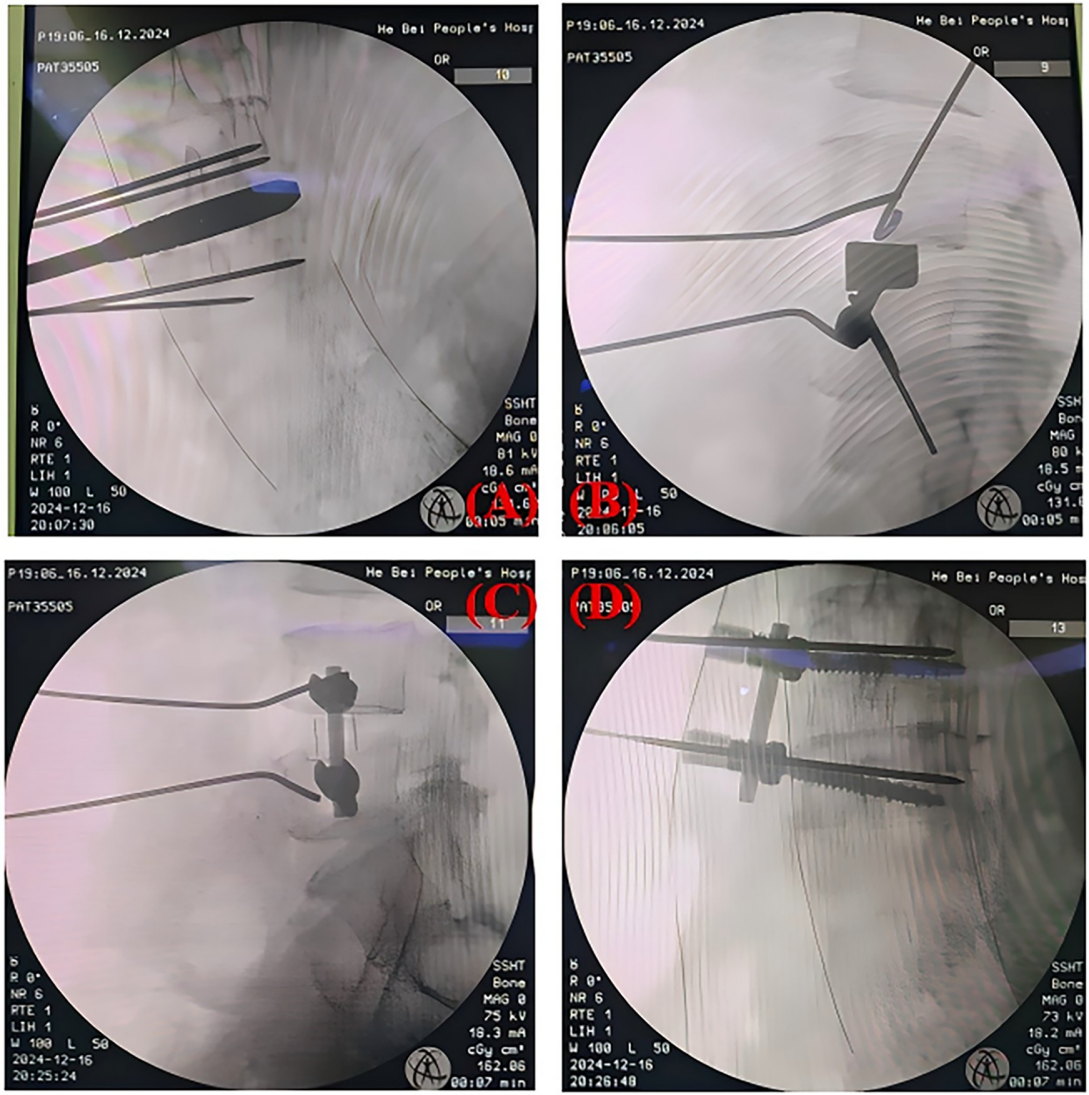
**(A,B)** show intraoperative fluoroscopic views after trial cage placement, while **(C,D)** display the final construct with implanted instrumentation.

A 6 cm oblique incision was made in the right anterolateral abdomen, extending posterosuperiorly to anteroinferiorly. The incision was carried through the layers of the anterior abdominal wall to enter the peritoneal cavity. Subsequently, the posterior abdominal wall was approached to expose the retroperitoneal structures. The transversalis fascia was not incised but instead bluntly dissected with finger dissection, sweeping the fascia and retroperitoneal fat anteriorly to naturally guide the approach toward the psoas muscle region. The psoas muscle was identified through blunt dissection of the anterolateral attachments to the intervertebral disc space.Simultaneously, the palpable pulsation of the abdominal aorta was identified. After locating the pulse, deeper finger dissection allowed identification of the non-pulsatile IVC, which presented as a soft tissue structure overlying the anterior disc space. Upon direct visualization through the incision, the IVC appeared deep blue with an approximate diameter of 2.5 cm, positioned anterior and slightly leftward relative to the spine. The oblique working corridor was established through simultaneous anterior mobilization of the peritoneal and vascular structures, coupled with posterior retraction of the psoas muscle. After adequate exposure of the target intervertebral disc, discectomy, cartilaginous endplate removal, and interbody cage implantation were performed. Given the oblique trajectory of OLIF, precise directional control is critical—deviation toward the spinal canal risks inadequate disc space penetration and potential neural injury. The retroperitoneal space was accessed via blunt dissection. After clearing superficial annular tissue, the annulus fibrosus was incised, followed by discectomy and intervertebral space preparation. Under fluoroscopic guidance, the disc space was appropriately distracted, and a properly sized interbody cage was implanted orthogonally. Supplemental lateral screw fixation was then performed (Figure 4). During retractor removal, meticulous hemostasis was verified to ensure no active bleeding remained. All residual bone graft material was meticulously cleared from the psoas muscle surface. The surgical site was copiously irrigated, followed by layered wound closure with placement of a closed suction drain.

### Postoperative outcomes

**Figure 5:**
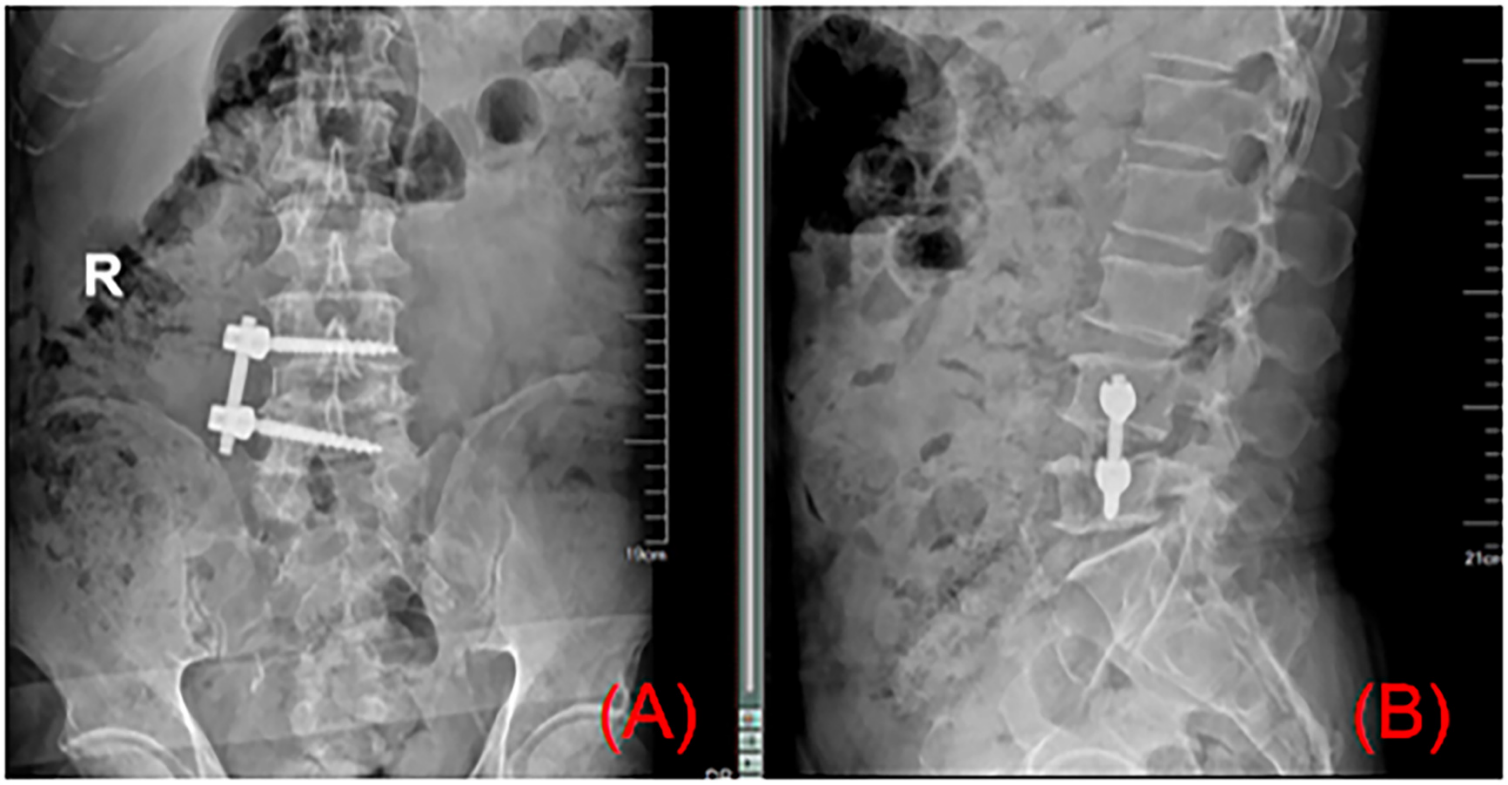
Postoperative radiographs **(A,B)** confirmed optimal positioning of the lateral screw-rod construct and interbody cage, consistent with intraoperative fluoroscopic findings.

The total operative time was 104 minutes, with an estimated blood loss of approximately 15 ml (Table 1). The position and size of the implant were confirmed by re-examination of the radiographs on the 3rd postoperative day (Figure 5). With the lower back pain VAS score decreasing from 7 to 2 and the lower limb pain VAS score decreasing from 5 to 1, the patient's pain was significantly relieved. Meanwhile, the patient's continuous walking distance gradually increased. No surgical complications were observed during the perioperative period (Table 2).

**Table 1:** Comparison of laboratory parameters between preoperative and postoperative periods.

Clinical laboratory tests	Preoperative	Postoperative (3 days)
WBC	9.97 × 10⁹/L	10.73 × 10⁹/L
RBC	5.4 × 10¹²/L	5.25 × 10¹²/L
Hb	14.8 g/dl	14.3 g/dl
Platelets	406 × 10⁹/L	394 × 10⁹/L
Procalcitonin (PCT)	0.053 ng/ml	0.101 ng/ml
Erythrocyte sedimentation rate, (ESR)	18 mm/h	24 mm/h
C-reactive protein, (CRP)	8 mg/L	32.72 mg/L

**Table 2:** Postoperative follow-up data demonstrated significant clinical improvement in patients.

Follow-up parameters	Preoperative	Postoperative (2 weeks)	final follow-up (7 months)
VAS (low back pain)	7	2	1
VAS (lower extremity pain)	5	1	1
ODI	58	34	16
walking tolerance	300 m	800 m	>1,000 m
Complications	–	No complications were observed	

Ostoperative telephone surveys were completed at 2 weeks and 7 months to evaluate recovery status.

## Discussion

In 2012, Silvestre et al. first reported the OLIF (10). This procedure accesses the target intervertebral disc through an oblique anterior abdominal incision. In the general population, the IVC is located anterior and to the right of the spine, so the surgical window is consistently chosen between the left edge of the AA and the right edge of the left psoas muscle. Molinares et al. (11) described the surgical window at the L2-S1 intervertebral discs through the dissection and measurement of 20 fresh-frozen cadaveric torso specimens. Through quantitative evaluation of the surgical window. Tao et al. (4) concluded that the positioning of major retroperitoneal vessels, particularly the IVC, is a significant potential factor contributing to variations in the surgical window. This is because intraoperative injury to the IVC can lead to catastrophic vascular complications. However, the above studies have not elaborated on the situation when anatomical variations in the IVC occur. Among the various anatomical variations described by Dilli et al. (12), both the left-sided IVC and the duplicated IVC are considered to influence the surgical approach (9, 13). Therefore, we report this case of OLIF performed via a right anterolateral approach, combined with the case reports from Berry (14) and Liu et al. (15), to provide references for the right-sided approach.For patients with scoliosis, prior lumbar surgery or significant abdominal scarring, a right-sided approach may be necessary.

This patient was treated with lateral screw-rod internal fixation. OLIF can be used alone without internal fixation (OLIF-SA), combined with lateral screw-rod internal fixation (OLIF-AF), and posterior percutaneous pedicle screw internal fixation (OLIF-PF) to obtain more stable support (16). OLIF-SA carries potential risks of complications due to its questionable biomechanical stability (17). The other two internal fixation methods differ in terms of perioperative complications and fusion rates. OLIF-AF has fewer complications because it does not require intraoperative repositioning or additional incisions. Compared to lateral fixation, OLIF-PF is widely used to enhance fusion rates (18, 19). Studies have shown that as the degree of osteoporosis increases, the range of motion in flexion-extension, lateral bending, and rotation decreases across all internal fixation methods (20). Compared with OLIF-PF, OLIF-AF has a larger range of flexion, extension, and rotation, and no significant difference in lateral bending range. Some advantages of OLIF-AF are well demonstrated in this case (Table 3). However, the long-term therapeutic outcomes must be evaluated through long-term follow-up data.

**Table 3:** OLIF with lateral fixation vs. Posterior Fixation: Similarities and Differences.

Similarities and differences	OLIF-AF		OLIF-PF
Similarities	1. This technique is designed to enhance spinal stability following OLIF and promote intervertebral fusion. 2. It should be used in conjunction with interbody cages to prevent cage subsidence or migration. 3. Indications include lumbar degenerative diseases (e.g., spondylolisthesis, discogenic low back pain, spinal stenosis). 4. Both techniques can be applied to osteoporotic patients. 5. With modern techniques, both procedures can be performed minimally invasively (e.g., percutaneous pedicle screw placement, small-incision approaches) to minimize soft tissue trauma.		
Key differences	Surgical approach	Requires additional posterior incision (midline/paramedian), potentially increasing trauma.	Completed through the same lateral OLIF incision, no additional approach needed (less invasive).
	Biomechanical properties	Provides 3-column fixation (anterior/middle/posterior), stronger against rotation/shear forces.	Primarily fixes anterior/middle columns, strong axial load resistance but slightly weaker in rotation.
	Stability advantages	Preferred for high-mobility segments (e.g., L4-L5) or complex cases (e.g., spondylolisthesis).	More suitable for mid-lower lumbar spine (L2-L5), optimal when posterior structures are intact.
	Operative time	Posterior instrumentation may prolong surgery (especially bilateral fixation).	Lateral fixation is simpler, typically shorter operative time.
	Radiation exposure	Requires repeated fluoroscopy for screw trajectory confirmation (higher radiation).	Fewer fluoroscopy shots needed (lower radiation exposure).
	Neurological risks	Posterior screws may risk nerve root/dura injury.	Lateral fixation requires avoiding lumbar plexus injury (e.g., femoral/genitofemoral nerves).
	Preferred indications	Severe spondylolisthesis • Multi-level fusion • Osteoporosis requiring rigid fixation.	1. Single-level degeneration 2. Cases without posterior decompression needs 3. Minimally invasive fast-track recovery.

### Patient-friendly version

Preoperative 3D imaging clearly demonstrated anomalous left-sided vascular anatomy, contraindicating the conventional approach. Although right-sided access represented a less common surgical strategy, it provided safer vascular avoidance. While minor incision-related tightness persists, the dramatic improvement in pain-related quality of life validates this approach.

At one-week follow-up, recovery met all expected milestones: ambulation was pain-limited only by surgical site discomfort, wounds healed without erythema or drainage, and oral analgesics provided adequate symptom control. The discharge protocol included detailed activity restrictions, red flag education, and scheduled 4-week postoperative evaluation.

## Conclusions

A left-sided IVC is uncommon but holds significant clinical importance. When the anatomical characteristics of IVC variants remain indeterminate, preoperative IVC venography becomes essential. This imaging modality provides definitive vascular mapping to guide safe surgical corridor selection, particularly for OLIF approaches in anatomically complex cases. Meanwhile, overcoming the muscle memory formed during the conventional left abdominal approach presents some challenges for surgeons. For this patient, the surgery via the right abdominal approach was successful. However, we are still following up to assess the long-term therapeutic outcomes.

## Data Availability

The original contributions presented in the study are included in the article/Supplementary Material, further inquiries can be directed to the corresponding author.
